# Diet in Disease

**Published:** 1893-03-18

**Authors:** Ernest Hart

**Affiliations:** Bachelier-ès-Sciences-es-Lettres (rèstreint), formerly Student of the Faculty of Medicine of Paris, and of the London School of Medicine for Women


### Diet in Disease.

By Mrs. Ernest Hart, Bachelier-ès-Sciences-es-Lettres (rèstreint), formerly Student of the Faculty of Medicine of Paris, and of the London School of Medicine for Women.

### XVI.—DIABETES (*continued*).

### Carlsbad, Marienbad, Vichy.

Carlsbad has been called "the hospital for diabetics." It is a hospital, however, of which the walls are the pine-clad hills, the roof the sunny sky, and the medicine the hot bubbling streams. At Carlsbad, the dietetic treatment of diabetics is carried out under the most agreeable and revivifying conditions.

The town of Carlsbad lies in a narrow, winding valley through which flows the stream of Tepel. The houses climb the hills on either side, and it seems to have been hard to find level spaces for the Curhaus, and concert and promenade rooms. At certain spots in the shallow river hot saline water is seen bubbling up through the crust of the earth and mixing with the stream. One of these hot springs, called the Sprudel, shoots high into the air, while others bubble up slowly into wells and reservoirs.

All the Carlsbad waters contain the same alkaline ingredients in almost identical proportions; they vary only from one another in the amount of carbonic acid they contain and in the temperature at which they come to the surface. This temperature varies from 100 deg. to 166 deg. The principal salts they contain are sulphate of sodium, chloride of sodium, carbonate of sodium, and carbonate of calcium. The springs are believed to be derived from a common source from seven to eight thousand feet below the surface of the earth. In fact Carlsbad may be considered to be set down over an immense cauldron of boiling water which is forced up under the pressure of the stream through cracks and holes in the earth's surface. The water is very agreeable to drink, and has been compared to rather salt chicken broth.

Life at Carlsbad is framed to be free of care, and as diabetes is frequently caused by over-anxiety of mind and the worry of life, it may be conceived that to be relieved altogether from the causes of the malady may alone effect the cure. On arriving at Carlsbad one is immediately visited by the tax collector, who demands a tax of from four to fifteen florins from every visitor according to his means. With the money obtained from this universal cure-tax, the immense forests which stretch for miles round about Carlsbad are maintained as a public garden; two bands of the best reputation, and composed of highly-trained performers, are engaged to give music to the visitors all day long, the theatre is subsidised, and the Curhaus and promenade rooms are kept up. Lodgings can be obtained at Carlsbad at every price. The usual course of life is as follows:—

Called early, the visitor is out in the fresh morning air by six o'clock at the latest, and with a glass cup suspended by a leather strap across the shoulder, he takes his way to the great Curhaus which covers the spouting waters of the Sprudel, or to the wells of the Schlossbrunn, Mühlbrunn, Elizabethqaelle, or Neubrunn, as directed by the physician. He joins the *queue* at the well, and in turn hands his cup to be filled by one of the neat little maidens, whose duty it is to charge the glasses. Waiting until the water is cool enough to drink, it is slowly sipped, while the delightful strains of Labitzky's band are enjoyed. A short walk of about twenty minutes is then taken either up and down the covered promenades of the Curhaus, or among the flower-beds of the Stadtpark. In the course of two hours three or four half-pints of hot water have been drunk, and three or four miles have been walked. By this time one feels not disinclined for breakfast, but rigid abstemiousness is the rule of life at Carlsbad, and though one might feel capable almost of eating a mutton chop or some ham and eggs or fish for breakfast it is not allowed. Cheerfully submitting, however, to the doctor's orders the many visitors are seen trooping into the various bakers' shops and purchasing their frugal breakfast in the form of a couple of "crescents," or some of the admirably made diabetic cakes. With these in a paper bag the patient betakes himself to one of the many little tables set out under the trees of the restaurant gardens, and breakfast is taken from coffee, the bread purchased, with perhaps a single boiled egg, or a few slices of German sausage.

After breakfast the papers of the day are leisurely read, and at about eleven o'clock the operation of bathing is gone through. The baths are of different kinds, hot saline baths and vapour baths; but the characteristic bath of Carlsbad is the peat bath. This consists of black peat pulverised, then screened and freed from accidental impurities and mixed with hot Sprudel water. After lying in this mixture for some time the patient takes a dip in a bath of clean water. The effect of the peat bath is said to be stimulating to the skin and sedative to the nervous system. The patient will then go home and rest for a while, till at about one o'clock the pangs of hunger become irresistible. He then betakes himself to one of the many large airy restaurants furnished with balconies or shady gardens, where he can take his midday meal in the open air. Dinner is frugal and strictly *Kurgemäss.* It may consist of a course of fish, meat, or fowl, the allowed green vegetables and cheese, with a single glass of lager beer or claret.

The afternoon is spent in strolling along the beautiful paths of the forest, or in walking through the valley to one of the gardens, where a delightful classical concert given by Labitzky's band may be enjoyed while sipping coffee under the shade of the trees.

The evening meal is taken about seven, and consists of a bowl of bouillon or a couple of poached eggs, or some dish making an equally light repast. For those who are well enough, and who can enjoy social pleasures, there is an excellent theatre and frequent dances at the Curhaus from eight to twelve, at which visitors are expressly requested to attend in *toilet de ville.* Most patients, however, seek their beds at about nine o'clock, having been sufficiently tired out by this idle day of drinking water, bathing, and taking pic-nic meals to the strains of an excellent band.

The influence of this life and the strict diet enforced on the diabetic patient is quite remarkable. It is ascribed by many to the waters; but it is, I believe, a fact that these waters may be taken at home where the patient is subjected to the usual worries, anxieties, and work of daily life, and they will not have the same result as when taken whilst living a quiet, open-air life, free of care, in Carlsbad. The cure is, in fact, not only a water cure, a bath cure, and a diet cure, but a music cure, a fresh air cure, and a *laissez-aller* life cure. In a very short time these beneficial influences are felt. The diabetic loses his dyspepsia, his depression of spirits, his parched lips, and his extreme thirst, and he begins to feel again that life can be enjoyed.

Careful daily examinations of the urine show at the same time that the percentage of sugar steadily diminishes; in fact in most cases of diabetes minor it will entirely disappear in the course of three or four weeks' treatment. The cure is, however, not a permanent cure; it is generally necessary for the patient to return to Carlsbad every year. In fact, the remembrance of freedom from his troubles becomes, as soon as they return the following year, persuasion enough to induce him to think of another visit to the springs. Men occupying responsible and important public posts have been known to be visitors at Carlsbad for twenty, thirty, and even forty years in succession, thus keeping their health and their possibilities of public usefulness and of personal enjoyment of life by means of an annual stay of three or four weeks at Carlsbad.

Marienbad is nineteen miles south of Carlsbad, and its waters closely resemble those of Carlsbad, excepting that they are of a much lower temperature, ranging from 43 deg. to 50 deg. Fahrenheit. The springs were first brought into notice in 1870 by the Abbot of the Convent of Tepel, and since then Marienbad has become a very fashionable resort, and is crowded in the summer with visitors. It is preferred by some to Carlsbad, owing to the fact that it is situated on a hill 2,000 feet above the sea level, instead of in the defiles of a narrow valley like Carlsbad. The air is fresh, in fact, at times, even chilly, but the beauty of the environs, the extent and variety of the walks through the forests, the general gaiety of the place make Marienbad one of the favourite spas of Bohemia.

Vichy is another spa which is greatly resorted to by diabetics. It is eight hours by rail south of Paris, and is situated in a pleasant valley 800 feet above the sea level on the bank of the river Allier. The waters are alkaline, and contain a large amount of bicarbonate of sodium. They vary in temperature. Some are hot, others nearly cold. The waters come from immense underground reservoirs, which may be visited by passing along dark underground passages. The effect of daily drinking these alkaline waters seems to be to maintain the fluids of the body in an alkaline condition, to promote oxidation and quicken tissue change, and improve assimilation. Thus they have the opposite effect of thinning the fat and fattening the thin; the former by improving digestion, increasing assimilation, and aiding the formation of flesh; the latter by rapidly oxidising inert and unnecessary fat tissue.

The influence of Vichy waters on diabetes is often very marked. In 100 cases treated by Barthey, 50 lost all traces of sugar; in 16 it was greatly diminished; while in 34 it remained stationary, although digestion was improved. Slight cases of diabetes improve greatly at Vichy, but more severe cases require protracted treatment and frequent return to the spa. The dietetic treatment is strictly enforced at Vichy, but there is much to amuse and distract the patient, and to make his period of "cure" pass pleasantly.

